# Deep learning networks find unique mammographic differences in previous negative mammograms between interval and screen-detected cancers: a case-case study

**DOI:** 10.1186/s40644-019-0227-3

**Published:** 2019-06-22

**Authors:** Benjamin Hinton, Lin Ma, Amir Pasha Mahmoudzadeh, Serghei Malkov, Bo Fan, Heather Greenwood, Bonnie Joe, Vivian Lee, Karla Kerlikowske, John Shepherd

**Affiliations:** 1 Department of Bioengineering, University of California-San Francisco Berkeley Joint Program, Room A-C106-B, 1 Irving St, San Francisco, CA 94143 USA; 2 Department of Radiology and Biomedical Imaging, UC-San Francisco, San Francisco, CA 94143 USA; 3 Kaiser Permanente Division of Research, Oakland, CA USA; 4 Accenture, San Francisco, CA 94143 USA; 5 Applied Materials, Santa Clara, CA USA; 6 Research Advocate, UCSF Breast Science Advocacy Core, San Francisco, CA 94143 USA; 7 Departments of Medicine and Epidemiology and Biostatistics, UCSF, San Francisco, CA 94143 USA; 8 Cancer Epidemiology, University of Hawaii Cancer Center, Honolulu, HI 96813 USA

**Keywords:** Breast Cancer, Masking, Mammography, Interval Cancer, Deep learning, Transfer learning, Neural network, Breast density

## Abstract

**Background:**

To determine if mammographic features from deep learning networks can be applied in breast cancer to identify groups at interval invasive cancer risk due to masking beyond using traditional breast density measures.

**Methods:**

Full-field digital screening mammograms acquired in our clinics between 2006 and 2015 were reviewed. Transfer learning of a deep learning network with weights initialized from ImageNet was performed to classify mammograms that were followed by an invasive interval or screen-detected cancer within 12 months of the mammogram. Hyperparameter optimization was performed and the network was visualized through saliency maps. Prediction loss and accuracy were calculated using this deep learning network. Receiver operating characteristic (ROC) curves and area under the curve (AUC) values were generated with the outcome of interval cancer using the deep learning network and compared to predictions from conditional logistic regression with errors quantified through contingency tables.

**Results:**

Pre-cancer mammograms of 182 interval and 173 screen-detected cancers were split into training/test cases at an 80/20 ratio. Using Breast Imaging-Reporting and Data System (BI-RADS) density alone, the ability to correctly classify interval cancers was moderate (AUC = 0.65). The optimized deep learning model achieved an AUC of 0.82. Contingency table analysis showed the network was correctly classifying 75.2% of the mammograms and that incorrect classifications were slightly more common for the interval cancer mammograms. Saliency maps of each cancer case found that local information could highly drive classification of cases more than global image information.

**Conclusions:**

Pre-cancerous mammograms contain imaging information beyond breast density that can be identified with deep learning networks to predict the probability of breast cancer detection.

## Background

Breast cancer is a common disease with 1 in 8 women experiencing some form of malignant breast cancer in their lifetime [1]. In 2013, this translated to approximately 230,000 new cases of invasive breast cancer and 40,000 deaths in the US alone [1]. Detecting and treating breast cancer is extremely important for women’s health and studies have shown that early detection of breast cancer yields higher survival rates [2].

Mammography is the current gold standard in screening for breast cancer in average-risk women. However, radiologically dense and complex tissue can reduce screening detection sensitivity leading to obscured breast lesions and cancers missed by screening mammography [3, 4]. These cancers discovered within 12 months after normal screening mammograms are defined as interval cancers, and the reduction of mammographic sensitivity from breast density is commonly called masking. Roughly 13% of breast cancers diagnosed in the U.S. are interval cancers [5], and identifying women at high risk of interval cancers could prove useful to inform discussions on supplemental imaging.

Previous studies have shown that the Breast Imaging-Reporting and Data System (BI-RADS) breast density and other quantitative density measures are not only risk factors for breast cancer, but also for interval breast cancer risk due to the masking effect of radiologically dense breasts [4, 6]. While clinically measured BI-RADS breast density is a risk factor for interval cancer such that federal and state legislation has been passed to notify women of high BI-RADS breast density [7], the classification is subjective and does not account for the texture of dense tissue [8–10]. Because of this, the American College of Radiology has asked for development of direct measures of masking and interval risk [11].

Researchers have also leveraged imaging methods beyond conventional 2D digital mammography such as tomosynthesis [12, 13], MRI [14], and diffusion weighted (DW) MRI [15–17]. Within DW MR imaging, improvements have been made in lymph node assessment and risk of recurrence. Additionally, computer vision methods have been applied to mammography to identify masking risk. Previous studies have measured the ability of pre-defined kernels and model observers to quantify masking and interval cancer risk, indicating some promise in computer vision to identify interval cancer risk [10]. Advanced computer vision methods such as deep learning have shown promise in many computer vision tasks and have performed extremely well in the ImageNet competition compared to traditional pre-defined kernel methods [18, 19]. Transfer learning [20, 21] of these networks has been effective in medical applications including breast cancer, where deep learning models were often able to equal or improve current classification or diagnostic schemes performed [22–27]. Another useful property of deep learning networks is their ability to highlight pixels containing unique information relevant to that image’s classification called saliency maps, which can be used in biological applications to develop hypothesis on the underlying biology or features associated with the classification of interest [26, 28].

Deep learning has consistently been applied to and improved on current diagnostic methods in many medical fields [21, 25, 29], from lung pathology diagnosis [26] to identifying diabetic retinopathy [30, 31]. Deep learning methods have also been applied to a wide variety of areas of breast cancer research with promising results [32]. Deep learning has been applied to improve lesion detection in computer-aided detection [24], to identify and segment soft tissue lesions [33, 34], to identify and reduce potential false positives to reduce biopsies [35], to effectively categorize the amount of dense tissue in mammograms [36], and to improve lesion classification systems on breast tomosynthesis images [27]. Our study further aligns with these studies in their goals of using deep learning to improve breast cancer outcomes.

The purpose of this study was to implement a deep learning network to investigate if unique imaging characteristics exist beyond breast density, to classify pre-cancerous mammograms that later result in either an interval or screen-detected invasive cancer within 12 months of the mammogram. We hypothesized that deep learning networks can more effectively quantify risk of interval cancer than BI-RADS breast density alone. If successful, these methods could be expanded to improve risk prediction models for interval cancer, develop automated methods or software that can aid radiologists in risk prediction, or to further understand radiomic quantities as they relate to underlying cancer biology.

## Methods

### Participants

Participants were selected from a screening population that had received full-field digital mammograms acquired from 2006 to 2015 from four radiology facilities, University of California – San Francisco, California Pacific Medical Center, Marin General Hospital, and Novato Community Hospital that participate in the San Francisco Mammography Registry. Ethics approval was obtained by the University of California – San Francisco Institutional Review Board for this retrospective analysis of mammograms. Invasive interval cancers from these institutions were included, defined as invasive cancers identified within 12 months of a negative screening examination. For interval cancers the negative mammogram prior to the mammogram leading to the eventual interval cancer diagnosis was chosen. An equal number of screen-detected cancers were matched by age and race if such matching data existed, based on all screen-detected cancers diagnosed at the four centers. Screen-detected cancers were defined as invasive cancers identified within 12 months of a positive screening examination. All mammograms were interpreted prospectively by radiologists during the course of routine clinical care. Cancers were identified by annual linkage to the state California Cancer Registry. Information was unavailable pertaining to the size of the lesions, specific cancer types, or whether the interval cancer was due to missed lesions or true interval cancers.

### Mammography

The de-identified raw, “For Processing” representation of the standard four screening views (Mediolateral-oblique (MLO) and Cranio-Caudal (CC) images of both sides) were used for this study. All images were acquired on Hologic Selenia full-field digital mammography systems. These images were pre-processed in order to maximize the information provided to the network in the following ways. First, the skin edge of these images was identified and excess background of the images was cropped out using thresholding and in-house software [37]. The images were then normalized on a 0 to 255 scale. Various methods exist to input images from multiple views, from inputting images individually, to making separate networks for each view, to combining the images as a collage [38]. We implemented a collage and the four views were stacked as a 2 × 2 collage image for each case, with one view in each quadrant. This allowed all four views to be contained in a single image. This method has been performed in applications such as brain MRI slices to indicate Alzheimer’s risk [38]. These images were then separated into a training and test set at an 80/20 split.

### Deep learning model

**Fig. 1:**
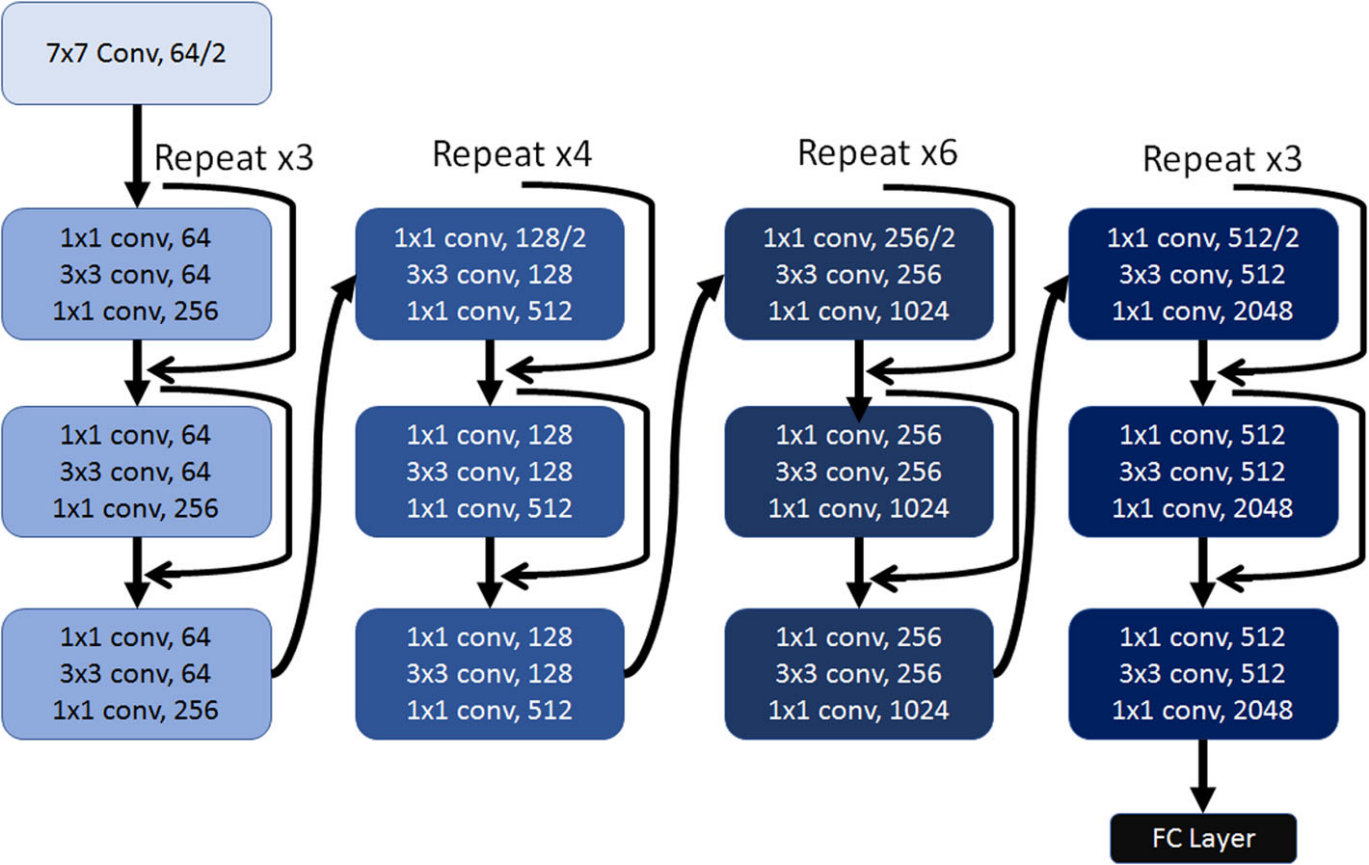
Schematic of the architecture of the deep learning network used in this study. YxY conv, M/N = M kernels of YxYx3 size and stride length of N (*N* = 1 if only M is listed). Fully Connected (FC) Layer = Dense (256), Dropout, Dense (1)

An existing deep learning network architecture (ResNet50) was implemented with ImageNet transfer learning weights on all convolutional blocks [19]. A fully connected layer was then added with 256 weights, a dropout layer, and a final weight with sigmoid activation to classify between screen-detected and interval cases. Figure 1 shows a diagram of the deep learning architecture and the fully-connected layer [19]. The weights of the fully-connected layer were randomly initialized and pre-trained on the training set images. During training, a binary cross entropy loss metric was optimized with a stochastic gradient descent optimizer. During model training, data augmentation was performed by introducing a random amount of shear, zoom, rotation, and horizontal and vertical reflection within specific ranges in order to increase the data variability and reduce overfitting. For each epoch, training loss, test loss, and accuracy were recorded and network weights were saved if it improved the test loss. The final network weights used were the weights with the best test loss throughout training.

Model hyperparameters for data augmentation, training parameters, and optimization parameters were selected through hyperparameter sweeps of a variety of hyperparameters. The hyperparameters were swept through a full realizable range of values for each parameter and loss and accuracy curves were examined to determine the range for each hyperparameter that resulted in a positive accuracy and loss trend as well as a small generalization gap between training and test data. Data augmentation hyperparameters were rotation, zoom, shear, horizontal reflection, and vertical reflection and were applied to the training data. Training hyperparameters were learning rate, batch size, number of epochs, image input size, and number of convolutional layers to allow to re-train weights. Model optimizer hyperparameters were momentum, regularization, and decay. Loss and accuracy were computed in the training and test set. Saliency maps were produced along with a contingency table enumerating the number of correct and incorrect predictions with some sample images in order to understand what factors contributed to incorrect and correct predictions. Training was done on an NVIDIA K2200 GPU with 16 GB RAM. Image preprocessing was done in Matlab r2015a (Mathworks, Natick, MA), ResNet50 was implemented with Keras and Tensorflow [39] using Spyder 3.2.3 and Python 3.5.

### Model statistical testing

After training was complete, conditional logistic regression was performed over the entire dataset in three cases: one with BI-RADS density as a classifier, one with the deep learning network predictions as a classifier, and one with both. In all cases interval vs. screen-detected breast cancer were the two outcomes. Receiver operating characteristic (ROC) curves were produced with area under the curve (AUC) values in all cases and compared. Statistical analysis and figure generation was performed via Spyder and R version 3.2.2.

## Results

A total of 316,001 examinations on were performed in the screening population, leading to a total of 245 interval cancers of which 182 women were available for this study. Table 1 shows the demographic information of the women from each case-type. These were matched by age and race to 173 women with screen-detected breast cancers. There were no screen-detected cancers that matched by age and race for 9 of the interval cancers. These were included in the deep learning training to maximize the dataset, but were excluded in the conditional logistic regressions to ensure matching. The descriptive statistics showed a lower body mass index (BMI) and higher proportion of women with dense breasts in the interval compared to the screen-detected breast cancer group. All other demographic and risk information was similar between groups.

**Table 1:** Descriptive statistics of the screen-detected and interval cancer groups. Percentage in each BI-RADS category are calculated excluding the missing/unknown groups

	Screen-Detected Group	Interval Group	*P*-Value
N	173	182	
Age, years (Standard Deviation)	57.8 (10.9)	56.8 (11.8)	0.28
BMI, kg/m^2^ (Standard Deviation)	24.9 (4.7)	23.5 (4.3)	< 0.0001
Time to Detection (Days)	56.3 (81.4)	239.8 (94.6)	< 0.0001
Race:			0.88
White	127	129	
African American	3	4	
Chinese	25	27	
Filipina	3	3	
Hispanic	0	2	
Japanese	5	8	
Mixed	5	5	
Other Asian	2	1	
Other Non-Asian	3	3	
Menopausal status	119 (69%)	123 (68%)	0.69
Family history of breast cancer	47 (23%)	60 (33%)	0.25
Previous history of breast biopsy	55 (32%)	68 (37%)	0.33
BI-RADS Frequency:			0.008
A: Almost Entirely Fatty	11 (7.8%)	3 (1.8%)	
B: Scattered Fibroglandularities	50 (35.5%)	33 (19.7%)	
C: Heterogeneously Dense	61 (43.3%)	78 (46.7%)	
D: Extremely Dense	19 (13.5%)	53 (31.7%)	
Missing Data	19	7	
Unknown	13	8	

Table 2 shows the end results of the hyperparameter sweep and optimal hyperparameters that were used in training our network. Of note we learned moderately aggressive image augmentation hyperparameters controlled overfitting while still allowing learning to take place. Additionally, a large batch size improved training by introducing the optimizer to more data and a learning rate in the range of 1e^− 3^ – 1e^− 5^ produced good learning results. High dropout in the final fully-connected layers helped to control overfitting as well. Optimal parameters were selected based on their ability to reduce overfitting based on the training and test loss. Training time on this system under these parameters was roughly 3 h per 500 epochs.

**Table 2:** Chosen hyperparameters with brief description. Hyperparameter sweep went through a realizable range for each hyperparameter and individual values were chosen to optimize training ability or to minimize overfitting, depending on the parameter

Hyperparameter (Range)	Hyperparameter Type	Interpretation	Chosen Value
Rotation (0–90)	Data Augmentation	Range for a random rotation	20
Zoom (0–1)	Data Augmentation	Range for a random zoom	0.5
Shear (0–1)	Data Augmentation	Range for a random shear	0.3
Vertical/Horizontal Flip (Yes/No)	Data Augmentation	Random chance of flip in respective direction	Yes/Yes
Momentum (0–1)	Optimizer Parameter	Accelerates or dampens oscillations in given direction.	0.3
Regularization (0–1)	Optimizer Parameter	Penalty applied to large image weights	0
Decay (0–1)	Optimizer Parameter	Learning Rate decay over each update.	1e-5
Dropout (0–1)	Fully-connected Layer	Percent of weights dropped out between dense layers in the FC layer.	0.95
Learning Rate (0–1)	Training Parameter	Importance attributed to weight updates.	1e-3
Epochs (Integer)	Training Parameter	Number of epochs performed	1000
Batch Size (2^n^ any n)	Training Parameter	Number of samples per gradient update	16
Image Size (Minimum 224)	Training Parameter	Input image size in pixels	224
nLayersRetrain (Fully Connected only – All Layers)	Training Parameter	Number of layers allowed to have their weights altered.	All Layers (173)

**Fig. 2:**
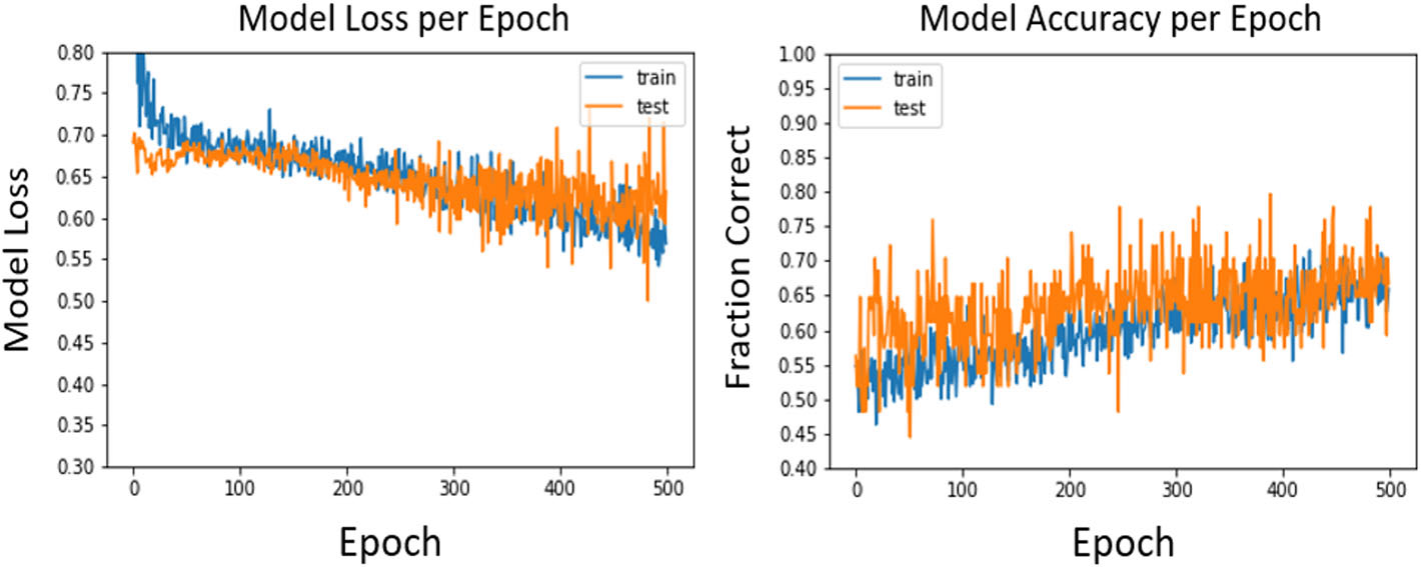
Loss and accuracy curves per epoch of the test and train set of the deep learning network. Best test loss occurred on epoch 482. At that epoch training loss and accuracy were 0.58 and 67.4%, respectively, and test loss and accuracy were 0.499 and 75.2%, respectively

Figure 2 shows the loss and accuracy of the model over time and compares the result of the training and test set. We can see that the generalization gap between train and test loss is small and that the curves in the test and train set are similar. This indicates that classification results were similar in both training and test sets. The best test loss occurred in epoch 482, with a test loss of 0.499 and test accuracy of 75.2%.

**Fig. 3:**
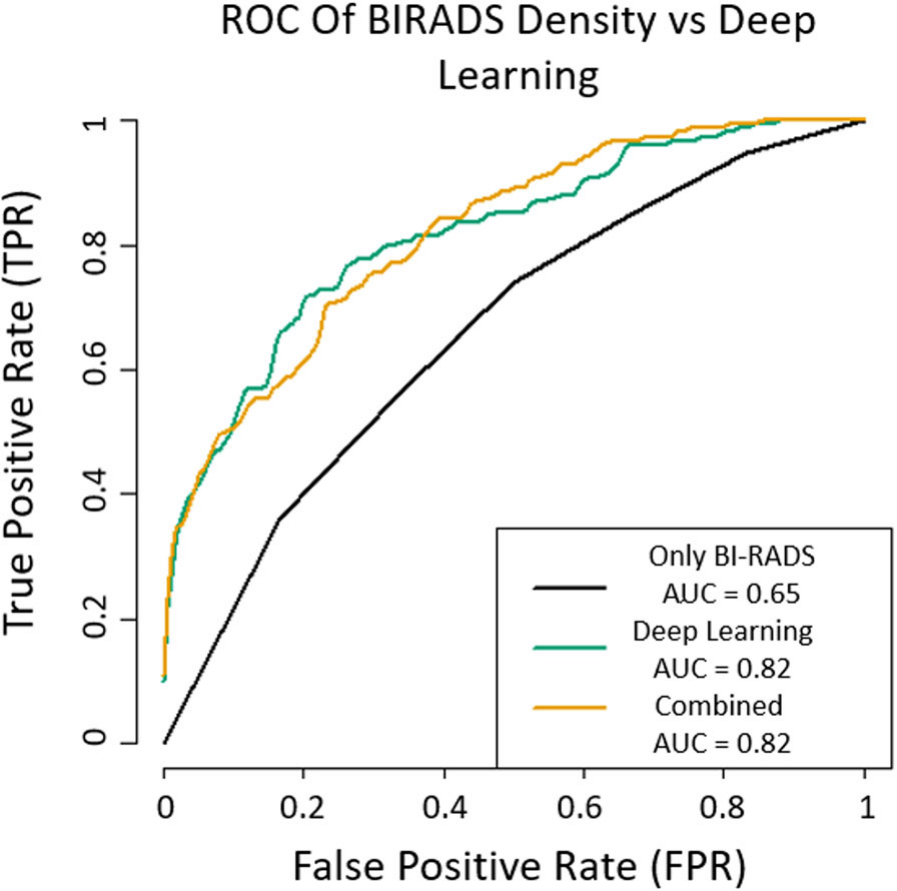
ROC Curves interval vs screen-detected cancer classification using BI-RADS density alone (Only BI-RADS) vs using the deep learning predictions (Deep Learning) vs using both as predictors (Combined). Prediction accuracy was 63% using BI-RADS density alone and 75% using deep learning alone

Figure 3 compares the classification ROC analysis and AUC of the deep learning network versus using just BI-RADS density in a conditional logistic regression, and a final analysis combining both of these methods. The deep learning network outperforms BI-RADS density alone in predicting interval versus screen-detected cancer.

Table 3 shows a contingency table quantifying the number and percent of correct and incorrect predictions in each category. The algorithm performed similarly for both cancer types - screen-detected and interval breast cancers were classified into their correct categories 77 and 74% of the time, respectively. Seventy-five percent of the total images (268/355) were correctly categorized.

**Table 3:** Contingency table of the number of correctly and incorrectly classified images from the deep learning network

Number (Percent)	Predicted Screened	Predicted Interval	Total
Actual Screened	134/173 (77.4%)	39/173 (22.5%)	173
Actual Interval	48/182 (26.4%)	134/182 (73.6%)	182
Total	182	173	

**Fig. 4:**
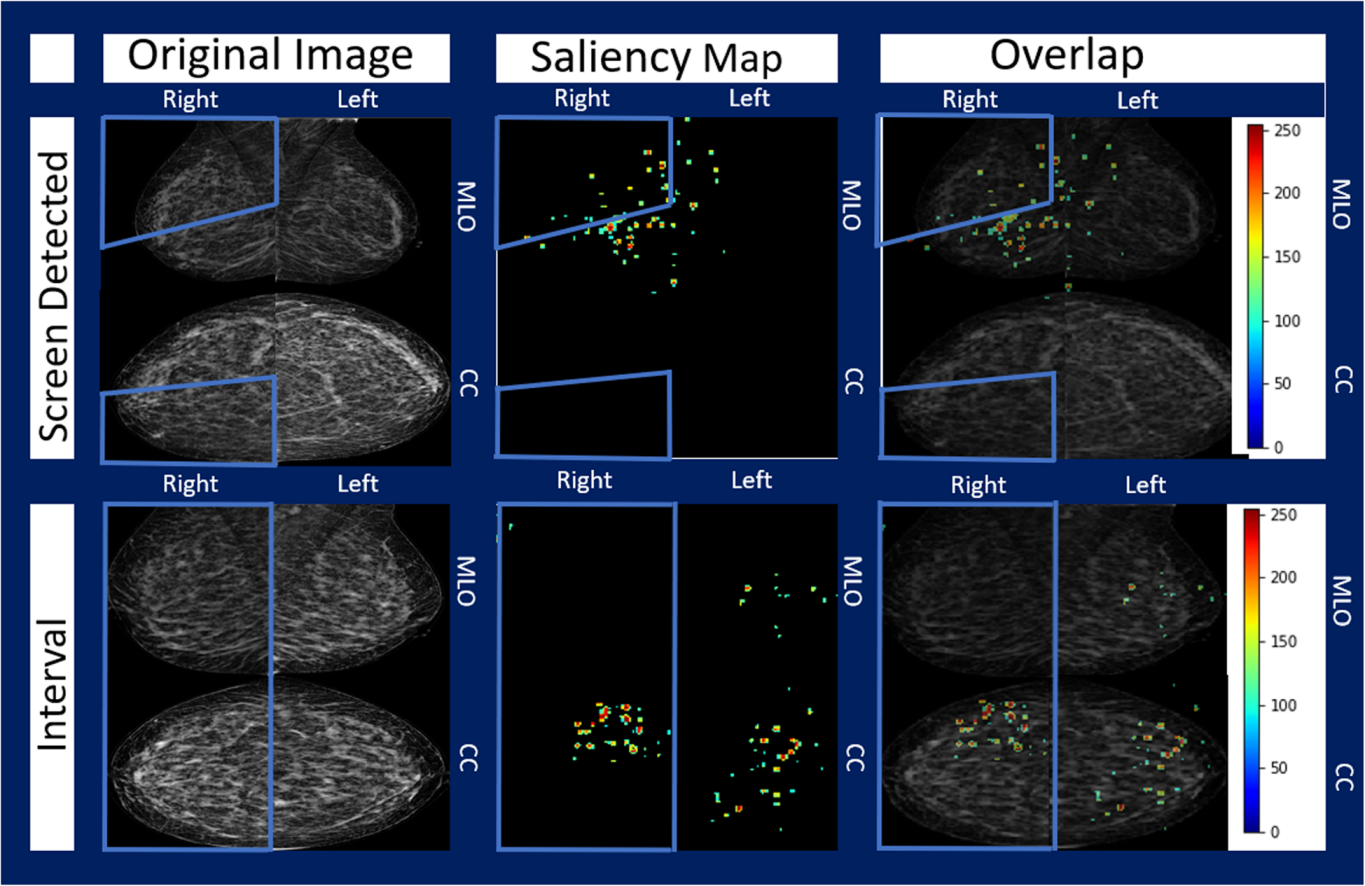
Saliency maps of sample screen-detected and interval images (both correctly classified). For each row, the pseudo-presentation images are shown (left) along with the saliency map (middle) that highlights the pixels that had above a 50% weight in classifying the image in its respective category (i.e. first row saliency map highlights weights that push towards decision of classifying as screen-detected decision). At right, the images are overlaid

Figure 4 shows the pseudo presentation mammograms (produced using methods described by Malkov, et al. [40], saliency maps, and then the superposition of the two for representative screen-detected and interval mammogram visits, both of which were correctly classified. The intensity of the saliency signal is shown from 0 to 255 color scale. A threshold was applied to highlight the regions above the 50% activation level in the network to improve image clarity. The side and quadrant (if available) where the cancer was found in subsequent mammograms is also shown. We observed that localized regions could highly influence the classification, but that broad regions of the breast could influence decision making as well.

## Discussion

We developed a deep learning algorithm that provided better discrimination than BI-RADS breast density for classifying interval cancer versus screen-detected cancer with a 75% classification accuracy compared with 63% for BI-RADS density. Deep learning networks have been applied in a variety of ways in breast cancer, but as of yet they have not been leveraged to identify risk of interval breast cancer. The results of our work indicate that a deep learning network is able to identify information in mammograms associated with interval breast cancer diagnosis that is not captured in the BI-RADS density classification alone.

Previous work by Kerlikowske et al. [4] showed that breast density was associated with increased prevalence of interval cancer in a screening population. Furthermore, Kerlikowske showed that using a combination of breast density and 5-year breast cancer risk to identify women for discussion about supplemental screening in the fewest women counseled per interval cancer occurrence. Recently, automated methods to quantify breast density have been shown to produce similar levels of interval risk as subjective BI-RADS density scores [6].

Other researchers have investigated radiomic features as a measure for interval risk. Strand et al. identified mammographic image features significant for interval breast cancer risk [9]. Holm et al. identified biological risk factors significant for interval risk after controlling for age and mammographic density [41]. Additionally, Mainprize et al. developed a direct measure of detectability that was significant for interval risk as well using model observers [10].

This study has several strengths. First, the dataset controls for age and race, helping to reduce possible confounding. Further, comparing our network to predictions based on BI-RADS density provides comparisons against current interval cancer risk factors [4, 7]. Additionally the transfer learning methods, the data preprocessing steps, data augmentation steps, and hyperparameter sweeps performed helped to maximize test accuracy.

Seventy-five percent of images analyzed were correctly classified, with slightly more actual interval images being misclassified compared to actual screen-detected images. This could be because the higher density of the interval images made them more difficult to classify. The saliency maps provided interesting information about the images and which regions influenced the decisions to classify the image as an interval or a screen-detected image. While it appears regions of interest in the interval image were related to density, further work must be done to examine how these regions in the saliency maps relate to the underlying biological and radiomic features of the image. The goal of saliency maps like this is to help bridge the gap between the deep learning network predictions and the radiologist or interpreter, helping to identify regions at high risk of interval breast cancer or identify regions to study further regarding why and how they contribute to interval breast cancer risk.

There were several limitations to our study. First, the computational limitations of our system required a large amount of downsampling, which likely lost significant imaging details and textures. Future work should utilize more powerful systems capable of dealing with larger image sizes. Additionally, because of the limited number of cases available and our goal to use as much data as possible in training, we were not able to employ a validation dataset and we included some interval images without direct matches or images without BI-RADS density values. We attempted to mitigate the risks from having a smaller dataset through transfer learning, data preprocessing and augmentation, and careful selection of hyperparameters. Additionally, comparing results of the test and train sets to ensure they had similar results helped to identify hyperparameter sets that produced good training and test results.

Another limitation in our dataset was that it did not control for BMI or length of time between mammogram or diagnosis, leading to potential bias between the mammograms. Additionally, this initial study only compared against the subjective BI-RADS density, and did not compare against more quantitative measures of breast density. Previous work has shown that BI-RADS density and automated density measures were shown to be similar risk factors for interval cancer [7], but future work should include comparisons against automated density measures and other known masking features. Further, we did not have information regarding cancer types or lesion sizes, which would be useful information for future analyses. An additional limitation was that this study did not include a healthy control group that did not develop cancer. Our hypothesis was that there were fundamental differences in the mammograms of women that develop interval versus screen-detected cancers. We found a strong signal to confirm this hypothesis, guiding the path to future studies that will compare interval and screen detected cancers to women that do not develop breast cancer. Lastly, we used a broad definition of interval cancer and did not differentiate between interval cancers from missed lesions and true interval cancers, which may have led to the deep learning network achieving some of its performance by detecting certain visible features. While many interval cancers occur because the lesion is masked, some interval cancers occur due to radiologist fatigue or error and others from fast growing lesions that develop after the previous mammogram [42]. Identifying and separating these subgroups can be difficult. We did not separate these types of interval cancers, which introduced additional noise into the dataset and may have weakened our results compared to using a dataset of only truly masked interval cancers.

## Conclusions

We conclude that pre-cancerous mammograms contain imaging information beyond breast density that can be used to predict the probability of breast cancer detection, and that deep learning models may be able to detect and identify that imaging information. This work could be expanded upon further to improve risk prediction models for interval cancer, develop automated methods or software that can aid radiologists in risk prediction, and understand if these deep learning predictions relate to underlying radiomic quantities or tissue biology.

## Data Availability

The datasets used and/or analyzed during the current study are publicly available from the corresponding author on reasonable request.

## Abbreviations

AUC: Area Under the Curve
BI-RADS: Breast Imaging – Reporting and Data System
BMI: Body Mass Index
CC: Craniocaudal
MLO: Mediolateral Oblique
ROC: Receiver Operating Characteristic
